# The establishment and characterization of the first canine hepatocellular carcinoma cell line, which resembles human oncogenic expression patterns

**DOI:** 10.1186/1476-5926-3-9

**Published:** 2004-11-26

**Authors:** Sacha Y Boomkens, Bart Spee, Jooske IJzer, Ronald Kisjes, Herman F Egberink, Ted SGAM van den Ingh, Jan Rothuizen, Louis C Penning

**Affiliations:** 1 Department of Clinical Sciences of Companion Animals, Faculty of Veterinary Medicine, Utrecht University, Yalelaan 8, P.O. Box 80154, 3508 TD Utrecht, The Netherlands; 2 Department of Pathobiology, Division of Pathology, Faculty of Veterinary Medicine, Utrecht University, The Netherlands; 3 Department of Infectious Diseases and Immunology, Division of Virology, Faculty of Veterinary Medicine, Utrecht University, The Netherlands

## Abstract

**Background:**

Hepatocellular carcinoma (HCC) is one of the most worldwide frequent primary carcinomas resulting in the death of many cirrhotic patients. Unfortunately, the molecular mechanisms of this cancer are not well understood; therefore, we need a good model system to study HCC. The dog is recognized as a promising model for human medical research, namely compared with rodents. The objective of this study was to establish and characterize a spontaneous canine tumor cell line as a potential model for studies on HCC.

**Results:**

Histomorphological, biochemical, molecular biological and quantitative assays were performed to characterize the canine HCC cell line that originated from a dog with a spontaneous liver tumor. Morphological investigations provided strong evidence for the hepatocytic and neoplastic nature of the cell line, while biochemical assays showed that they produced liver-specific enzymes. PCR analysis confirmed expression of ceruloplasmin, alpha-fetoprotein and serum albumin. Quantitative RT-PCR showed that the canine HCC cell line resembles human HCC based on the measurements of expression profiles of genes involved in cell proliferation and apoptosis.

**Conclusions:**

We have developed a novel, spontaneous tumor liver cell line of canine origin that has many characteristics of human HCC. Therefore, the canine HCC cell line might be an excellent model for comparative studies on the molecular pathogenesis of HCC.

## Background

Hepatocellular carcinoma (HCC) is one of the most worldwide frequent primary tumors in man, with an estimated 564,000 new cases and almost as many deaths in 2000 [1]. It almost always develops in the setting of chronic hepatitis or cirrhosis, conditions in which many hepatocytes are destroyed, inflammatory cells invade the liver, and connective tissue is deposited. Unlike colorectal carcinoma, for example, for which a model can be generated based on known molecular events occurring during the process of carcinogenesis [2], the pathogenesis of HCC is largely unknown [3]. Although many risk factors have been reported to be involved in the transformation from a normal cell into a malignant tumor cell, such as HBV, HCV, alcohol, aflatoxin B, cirrhosis, older age, and male gender, the molecular mechanisms of neoplastic transformation and progression in HCC are not yet well understood.

However, the study of those mechanisms is hampered because the liver tissue of patients with HCC has only limited value and primary hepatocytes are difficult to maintain in culture. Furthermore, primary hepatocytes rapidly lose detoxifying P450 isoenzymes. In addition, and because of the heterogeneity of the molecular genetic changes that can lead to HCC across species, molecular genetic studies in animals have not yet provided a precise general model for the molecular pathogenesis of HCC in humans. The dog is a valuable model for human comparative studies, since it has a comparative life span and habitat and thus similar risk factors and its domestication started over 10,000 years ago [4]. Moreover, and like rodents, the dog develops spontaneous hepatocellular tumors. However, these tumors are not associated with hepatitis and cirrhosis, and develop in normal livers. Furthermore, the entire genome of the dog is currently being sequenced, what will allow further detailed species-specific molecular analyses.

Here, we describe the establishment and morphological, immunohistochemical, biochemical, and molecular characterization of the first canine hepatocyte cell line derived from a spontaneous HCC of a dog. The objective of this study was to investigate whether this cell line could be used as a potential model for studies on human HCC. Therefore, we investigated whether this canine hepatocyte tumor cell line had features similar to human HCC with respect to mutations in the hepatocyte growth factor receptor (c-MET) gene and the differential gene expression of several oncogenes, proto-oncogenes and proteins involved in proliferation, apoptosis and cell survival.

## Results

### Histopathology of the donor dog

**Figure 1:**
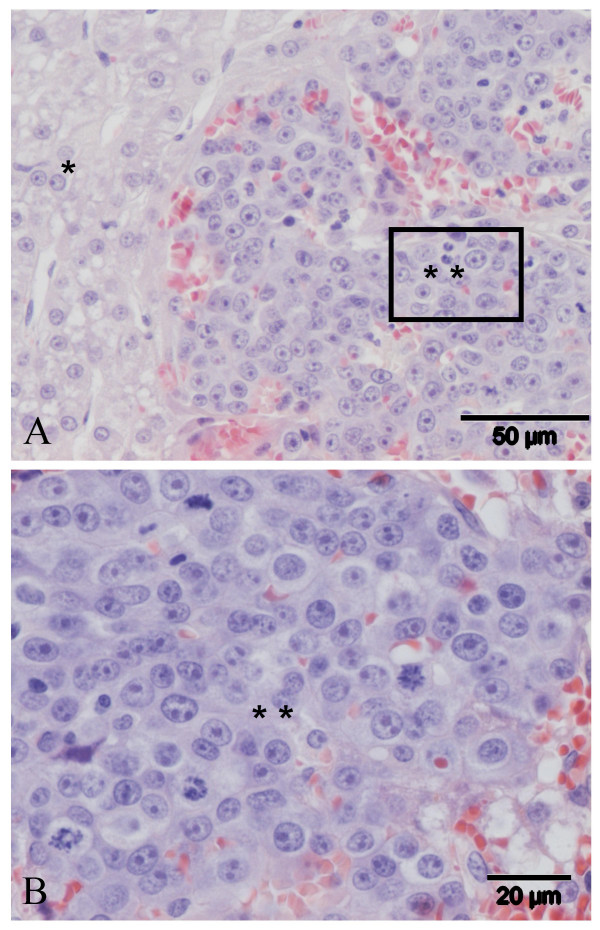
Histopathological characteristics of the original liver tumor from which the cHCC cell line is derived. **A**) A well-defined tumor area (*), as well as a carcinomatous area (**); **B**) An enlargement of the carcinomatous area (**).

The primary neoplasm was histologically characterized by broad trabeculae, 2 – 6 cells in thickness, of well-differentiated hepatocytes and separated by sinusoidal structures lined by endothelium. The hepatocytes had uniform moderately sized nuclei and small nucleoli; mitotic figures were very rare. Regularly areas with marked steatosis or glycogen accumulation within the neoplastic hepatocytes were observed. Locally within this well differentiated tumor there was a carcinomatous area characterized by broad trabeculae of more basophilic cells with large nuclei, moderate anisokaryosis, usually one or more large nucleoli and 3–5 mitotic figures per high power field (Figure 1). The non-affected liver histology of the donor dog showed no abnormalities, such as inflammation.

### Development and histomorphological characterization

Directly after establishment of the initial cell suspension (June 19, 2002), the cells appeared pleiomorphic. After approximately 10 weeks of culturing, the cells formed clusters as rounded, vital cells, which are non-adherent. This characteristic has remained ever since. Freezing and re-culturing of the cell line had no effect on cell growth. A 1:10 splitting and medium refreshment of the culture by careful trypsinization once a week (a "passage") is optimal. The tryspinized cell clusters were further cultured with fresh DMEM culture medium. The cells rather grow in these smaller clusters and not as single cells.

**Figure 2:**
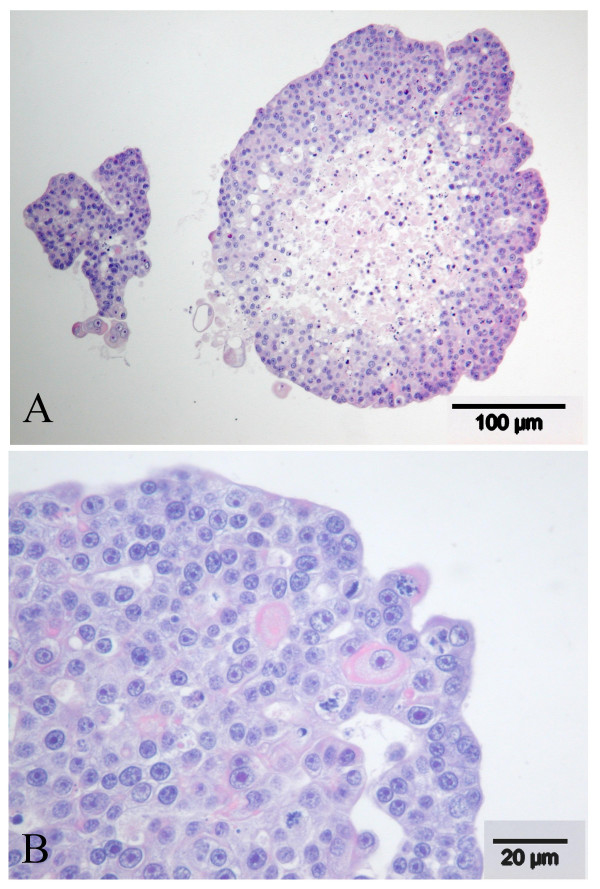
Histomorphological characterization cHCC cell line. **A**) Solid cell clusters of large epithelial cells with papillary projections at the periphery and extensive central necrosis. Bouin fixation, HE staining; **B**) Papillary growth of moderately pleiomorphic cells with anisocytosis and anisokaryosis, prominent nucleoli, and multiple mitotic figures. Carnoy fixation, HE staining.

**Figure 3:**
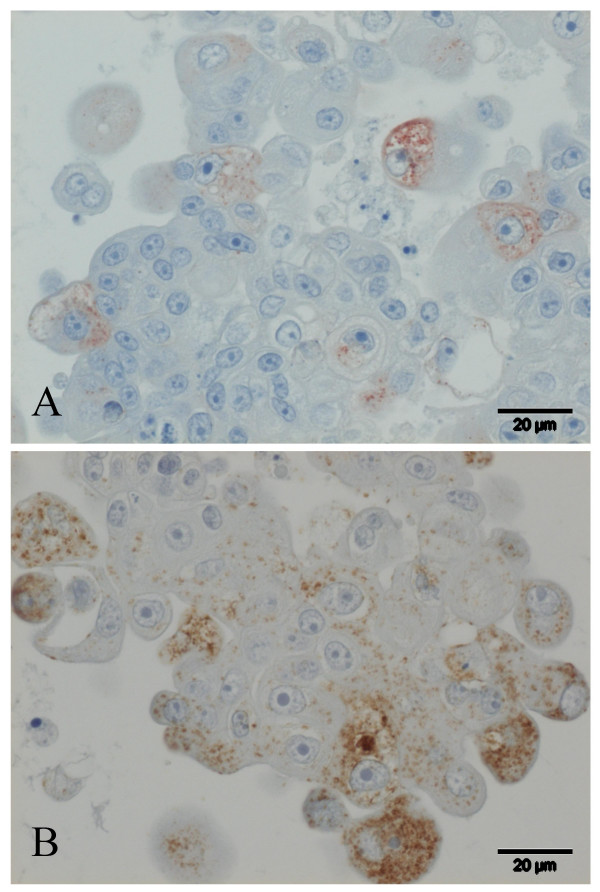
Immunohistochemical characterization cHCC cell line. **A**) Immunohistochemical staining for HepPar1. Moderate granular cytoplasmic staining in the majority of the cells. Bouin fixation; **B**) Immunohistochemical staining for CK7. Slight to moderate granular cytoplasmic staining in a minority of the cells. Bouin fixation.

The cell clusters were collected, fixed and handled as described. As shown in Figure 2, histology revealed solid cell clusters of large epithelial cells, with papillary projections at the periphery and extensive central necrosis. The cells were polygonal and moderately pleiomorphic, 10 – 20 μm in the largest diameter, showed marked anisocytosis and sometimes vacuolation of the cytoplasm. The nuclei were large (5–10 μm) and centrally located, with large and often multiple prominent nucleoli, and many, sometimes bizarre, mitotic figures (Figures 2A and 2B). Immunohistochemical staining for both HepPar1 and CK7 was best after Bouin fixation. In the Bouin fixed material, the hepatocyte marker HepPar1 (Figure 3A) revealed moderate granular cytoplasmic staining in the majority of the cells; CK7 (Figure 3B) showed slight to moderate granular cytoplasmic staining in a minority of the cells. As controls for the two stainings, liver tissue and kidney tissue of a healthy dog was used. These were positively and negatively stained, respectively. HepPar1 staining shows throughout the liver tissue samples, whereas the CK7 is localized in the bile ducts.

### Biochemical characterization

The activity of ALT, GLDH and AST was measured to investigate whether the cHCC cells produced liver characteristic enzymes. In order to compare the amount of hepatic enzymes produced by the cHCC cell line, those measurements were also performed for the widely used human hepatocyte cell line HepG2 (enzyme activity of HepG2 was set at 100%), and the commonly used canine kidney cell line MDCK. The results showed the cHCC cell line produced 25% of the highly liver-specific ALT compared to HepG2, whereas the MDCKs did not produce ALT at all (Table 3). Of another specific liver enzyme, GLDH, the cHCC cell line produced 19% of the activity of HepG2, whereas the MDCK cells produced only 10%. The cHCC cell line produced 28% of AST compared to HepG2, whereas MDCK produced only 8%.

**Table 3:** Liver enzyme activity measurement of the cHCC cell line compared with HepG2 and MDCK.*

	**ALT (%)**	**AST (%)**	**GLDH (%)**
**HepG2**	100	100	100
**cHCC**	25.8	27.7	19
**MDCK**	0	8	10

* Note: The activity of the enzymes is given as a percentage compared to the activity of proteins in HepG2.

### Molecular characterization

To further examine whether the cell line truly consists of hepatocytes, we isolated RNA from the cHCC, made cDNA, and performed PCRs for the gene expression of hepatocyte markers. The cHCCs proved to be PCR-positive for canine serum albumin, alpha-fetoprotein and ceruloplasmin. All obtained products were sequence confirmed.

### Mutations in the c-MET gene

**Figure 4:**
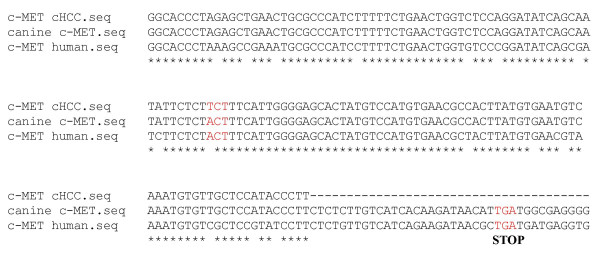
Mutation in the c-MET gene of cHCC (in figure as "c-MET cHCC") compared to healthy liver tissue (canine c-MET; Gen Bank accession number AB118945; "canine c-MET" in figure) and human c-MET (Gen Bank accession number NM_000245; "c-MET human" in figure) sequences. nt 4021–4219 of the canine c-MET is shown. The mutation is marked in grey at nt 4089 (A to T) of the canine c-MET, which corresponds to a change of amino acid 1363, from a threonine to a serine (T1363S). The STOP codon in the canine and human c-MET is also marked in grey. Alignment was performed by SECentral CLUSTAL W (1.7) multiple sequence alignment.

Mutations in exons 15–21 of the c-MET gene have been described in human HCC. A PCR was therefore performed with primers based on this region of the c-MET gene. The products were analyzed and aligned with known canine and human c-MET sequences (Gen Bank Accession numbers AB118945 and NM_000245, respectively). At nucleotide position 4089, a thymine (T) instead of an adenine (A) was observed, which resulted in a serine in the cHCC cell line versus a threonine in healthy tissue at codon 1363 (T1363S) (see Figure 4).

### Quantitative measurements of mRNA levels of gene products differentially expressed in cHCC

**Figure 5:**
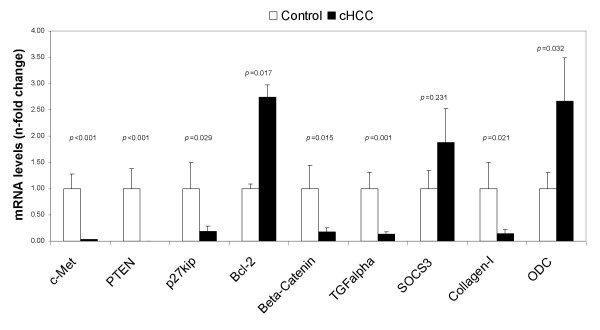
Differential gene expression profiles of the cHCC cell line as compared to liver tissue of healthy dogs, measured by quantitative Real-Time PCR. Data represent mean ± SE of the groups. The "n" for these figures stands for the fold change of the gene expressions of the cell line as compared with our control group.

To explore whether the cHCC cell line has similar expression profiles of genes involved in neoplastic growth and apoptosis as human HCC, the mRNA levels of the following genes were measured by means of quantitative RT-PCR: c-MET, PTEN, p27kip, Bcl-2, beta-catenin, SOCS3, ODC, TGF-alpha and collagen-1. The expressions of these genes were normalized by relating them to the housekeeping genes, HPRT and beta-actin. As shown in Figure 5, c-MET, a receptor tyrosine kinase involved in cell survival and growth, was down-regulated 33-fold compared to the control group. PTEN, an inactivator of the Akt/PKB pathway was down-regulated over 200-fold. To further substantiate activation of Akt/PKB, we measured two downstream targets, p27kip and Bcl-2. They were indeed down-regulated 5-fold and up-regulated 3-fold, respectively. HGF, the ligand of c-MET, induces mRNA levels of beta-catenin and TGF-alpha, both involved in cellular growth. The latter two were down-regulated 6- and 7-fold, respectively. From two novel proteins associated with hepatocellular carcinomas in man [5], suppressors of cytokine signaling type 3 (SOCS3) and collagen-I, a non-significant 2-fold elevation of SOCS3, were found and a 7-fold, significant down-regulation of collagen-I was observed. The gene expression of a proliferation factor, ornithine decarboxcylase (ODC), also proved to be elevated 3-fold.

## Discussion

We have described the establishment and characterization of a canine hepatocyte tumor cell line, derived from a spontaneous HCC in a dog. Both immortalized hepatocytes and hepatic progenitor cells come from various transgenic mouse lines [6], are drug-induced [7], or are obtained after SV40 large T-antigen transfection [8]. However, immortalization with SV40 induces HGF/c-MET activation via an autocrine HGF loop [9]. This clearly contrasts with the cHCC, where HGF-induced growth is absent (data not shown), most likely because of severely reduced c-MET levels.

Our morphological study has accumulated good evidence – but no definitive proof of the hepatocytic and neoplastic nature of the cHCC cells. Positive staining for hepatocyte marker HepPar1 strongly indicates the hepatocytic origin of the cultured cells [10]. The neoplastic nature of the cells can be deduced from the pleiomorphism of the cells, the number of sometimes bizarre mitotic figures. The simultaneous presence of CK7 and HepPar1 positive cells is also consistent with the neoplastic nature of the cells [11], and suggests the presence of both fully differentiated hepatocytes and progenitor cells in the cHCC cell culture. The hepatocytic nature of the cHCC cell line is further indicated by the activity of the liver-specific enzyme ALT and the expression of hepatocyte markers, like serum albumin, ceruloplasmin, and alpha-fetoprotein, as it is also the case for the human tumor liver cell line HepG2.

Binding of HGF to c-MET triggers tyrosine autophosphorylation of the intracellular domain in the c-MET receptor and induces responses that account for mitogenesis and growth. In human c-MET, point mutations have been described in the tyrosine kinase domain, which may be associated with the development of primary liver carcinomas [12]. In our study, we detected an unknown point mutation near the tyrosine kinase domain, which results in a conserved change from a threonine to a serine. Whether this mutation has any influence on the autophosphorylation of two adjacent tyrosines [13] remains to be proven.

In quantitative RT-PCRs, we measured the differential gene expressions of several gene products involved in proliferation/growth and cell survival. In human HCC, the c-MET gene-expression was observed to be induced in 60% of the cases [14], whereas we found a down-regulation of the c-MET gene-expression. Although we only measured mRNA levels of c-MET, we can correlate them to their protein expression levels [15]. Furthermore, lack of HGF-responsiveness was also observed by reduced expression of beta-catenin and TGF-alpha in the cHCC cell line, which is in accordance with findings in human HCC [16].

It has also been observed that the tumor-suppressor PTEN, the Akt/PKB pathway inhibitor [17], was down-regulated drastically, leading to an increased activity of Akt/PKB, as shown by the anti-apoptotic protein Bcl-2 and the cell-cycle inhibitor p27kip, which were induced and inhibited, respectively. Both p27kip and PTEN are inhibited in human HCC as well [17,18]. In addition, the gene expressions in cHCC of SOCS3 and collagen-I were elevated (although not significantly) and inhibited, respectively, as it was also detected in human HCC [19]. Moreover, we found an up-regulation for ODC. This proliferation factor, induced by several growth factors, is responsible for proliferation in many cell types [20]. Taken together, the expression data show elevated proliferation, increased cell survival, and reduced apoptosis, which explains the neoplastic nature of cHCC.

## Conclusions

From the morphological, biochemical, and molecular biological assays performed in this study, we conclude that the cHCC cell line clearly represents hepatocytes. In addition, cHCC has neoplastic characteristics comparable to HCC in man. Therefore, this cell line can be used as a model not only to study the molecular pathogenesis of human HCC, but also to investigate possible etiological agents of canine hepatitis.

## Methods

### Donor dog

Our material was taken from the liver of an eleven-year old, privately owned female Cairn Terrier dog, diagnosed with HCC by histological analysis. With the owners' consent, the dog was routinely anesthetized, parts were taken from various areas of the neoplastic liver tissue. In addition, liver tissue was collected for histological analysis, fixed in 10% buffered formalin and paraffin embedded. Sections were routinely stained with hematoxylin and eosin (HE). Then, the dog was immediately euthanized.

### Isolation and culturing conditions

Immediately after resection, the liver samples were kept in DMEM culture medium supplemented with 10% fetal calf serum (FCS; Fetal Calf Serum Gold, PAA Laboratories GmBH, Pasching, Austria), penicillin and streptomycin (P/S; 100 IU/ml and 100 μg/ml final concentration, respectively) and were kept on ice. Under sterile conditions, liver samples of various areas were cut into small pieces (5 × 5 mm) and trypsinized with 30 ml of trypsin/EDTA (0.5 g/l trypsin 1:250 and 0.2 g/l EDTA; BioWhittaker Europe, Verviers, Belgium) in a sterile Erlenmeyer flask placed on a stirring platform for 30 minutes. The cell suspensions were filtered with a 70 μm nylon filter (Falcon; Becton Dickinson Labware, Franklin Lakes, NJ). Erythrocytes were lysed from the filtered suspension. The remaining cell suspension was resuspended in DMEM supplemented with 10% FCS and P/S and was cultured at 37°C with 5% CO_2 _and 95% air under a humidified atmosphere in non-coated flasks. It was observed every day for any changes. The medium was refreshed twice a week.

### Histomorphological characterization

After 3 weeks of culturing with medium refreshment only and no splitting of the culture, the content of a T80 cm^2 ^flask with the cHCC cell culture was harvested by transferring the entire contents of the flask to a tube, which was centrifuged for 10 minutes at 1,500 g. The supernatant was replaced by freshly made fixation fluid for 4 hours. For optimal immunohistochemical staining, four different fixatives were used: zinc sulfate formalin, Bouin, Carnoy, and 10% neutral buffered formalin. The fixated cell pellet was transferred to a foam leaf-protected plastic embedding cassette. After fixation, samples were manually dehydrated and embedded in paraffin. Sections (3 μm thick) were cut and stained with hematoxylin and eosin (HE). For immunohistochemical staining, paraffin sections were mounted on poly-L-lysine coated slides, post-fixed into ice-cold acetone fixation fluid for 10 minutes, air dried and stored at room temperature (RT) until use.

For the detection of HepPar1, slides were deparaffinized, immersed in 10 mM Tris, 1 mM EDTA buffer (pH 9), heated in a microwave oven for 10 minutes for antigen retrieval, cooled down for 10 minutes at RT and washed in PBS buffer. Endogenous peroxidase activity was blocked by 0.3% H_2_O_2_, in methanol, for 30 minutes at RT. After washing with PBS buffer containing 0.1%Tween-20, background staining was blocked by incubating the sections with normal goat serum (1:10 diluted in PBS), for 30 minutes. Sections were incubated overnight at 4°C with the primary antibody HepPar1 (clone OCH1E5, Dakocytomation, Glostrup, Denmark) diluted 1:50 in PBS. After washing in PBS-Tween, slides were incubated in DAKO EnVision™ + reagent, HRP-labeled (Dakocytomation,) for 45 minutes at RT. After washing in PBS buffer, sections were developed using 3,3-diaminobenzidine as chromogen, and counterstained with hematoxylin.

For the detection of CK7, the slides were treated as described above but without antigen retrieval, and incubated overnight at 4°C with mouse anti-human CK7, clone OV-TL 12/30 (Dakocytomation), diluted 1:25 in PBS with 1% bovine serum albumin. For both HepPar1 and CK7, formalin-fixed paraffin-embedded canine liver and kidney tissue controls were incubated with and without the primary antibody. In contrast to the cell culture, in the liver tissue antigen retrieval for CK7 was necessary and, therefore, a 40-minute proteinase-K (Dakocytomation) digestion at RT was performed before blocking of the endogenous peroxidase, in methanol.

### Biochemical characterization

The content of a T80 cm^2 ^flask with the cHCC cell culture was harvested, spun down for 5 minutes at 1,500 g, the cell pellet was washed in 10 ml PBS, centrifuged for 5 minutes at 1,500 g and the cells were resuspended in 1 ml PBS. Two hundred μl of the cell suspension were lysed and homogenized with a pestle in RIPA buffer containing 1% Igepal, 0.6 mM phenylmethylsulfonyl fluoride, 17 μg/ml aprotinine and 1 mM sodium orthovanadate (Sigma Chemical Co., Zwijndrecht, The Netherlands), for 30 minutes on ice. Total protein concentrations were calculated using a Lowry-based assay (DC Protein Assay, BioRad, Veenendaal, The Netherlands).

For the liver enzyme measurement, 800 μl of the cell suspension was centrifuged at 12,100 g for 5 minutes. The pellet was lysed in milliQ by vortexing, centrifuged again for 5 minutes at 12,100 g and the supernatant was analyzed in a Beckman Synchron CX7 analyzer. The following enzymes were measured: ALT, AST and GLDH. AST and ALT were measured at 37°C with the Tris-pyridoxal phosphate method with Beckman-Coulter reagent. GLDH was measured with Roche reagent. All samples were subjected to the external quality control mission of the Dutch Foundation for Quality Assessment in Medical Laboratories.

As comparison, the widely used human hepatoma cell line HepG2 (Deutsche Sammlung von Mikroorganismen und Zellkulturen GmbH, DSMZ, Germany) and MDCK canine kidney cell line (own collection) were used. These were grown to 80–100% confluency (T80 cm^2 ^flask) under standard culturing conditions, as described for the cHCC cell line.

### RNA isolation and reverse-transcription PCR

Total cellular RNA was isolated from each frozen canine liver tissue in duplicate and from all the cell cultures used in this study using the Qiagen RNeasy Mini Kit (Qiagen, Hilden, Germany) according to the manufacturer's instructions. The RNA samples were treated with DNase-I (Qiagen RNase-free DNase kit). In total 3 μg of RNA was incubated with poly (dT) primers at 42°C for 45 min, in a 60 μl reaction, using the reverse transcription system (Promega Benelux, Leiden, The Netherlands).

### Molecular characterization

To examine whether the cell line consisted of hepatocytes, we isolated total RNA and made cDNA as described above. PCRs were performed to investigate the gene expressions of hepatocyte (albumin, alfa-fetoprotein, ceruloplasmin) markers. All reactions were performed in a 50 μl volume with a thermal cycler (MJ Research Inc., Watertown, MA). Reaction mixtures contained 0.2 μM of each oligonucleotide primer (Isogen Life Science, Maarssen, The Netherlands), PCR buffer (Invitrogen Corporation, Carlsbad, CA), 2.5 U of Platinum *Taq *polymerase (Invitrogen), 2 mM MgCl_2 _(Invitrogen) and 250 μM of each nucleotide (Promega Corporation, Madison, WI). The PCR conditions were: initial denaturation at 95°C for 4 min, followed by 40 cycles consisting of denaturation at 95°C for 1 minute, annealing at 60°C for 1 minute, elongation at 72°C for 1 min, and, finally, an elongation step at 72°C for 10 min. The PCR products were analyzed on a 1.5% agarose gel, and the DNA fragments were visualized with ethidium bromide. The primers used for these PCRs are depicted in Table 1.

**Table 1:** Oligonucleotides for RT-PCR used in this study.

**Primer**	**Target**	**Primer sequence (5'-3')**
mutMETF1	C-terminal part of canine cMET	CCT TGG AAA AGT AAT AGT TC
mutMETR1	C-terminal part of canine cMET	GTT TCA TGT ATG GTA GGA C
mutMETF2	C-terminal part of canine cMET	GAA GTT TCC CAG TTT CTG AGC
mutMETR2	C-terminal part of canine cMET	AAG GGT ATG GAG CAA CAC AT
CSA U	Serum albumin	GTT CCT GGG CAC GTT TTT GTA TGA
CSA L	Serum albumin	CTT GGG GTG CTT TCT TGG TGT AAC
Ceruloplasmin U	Ceruloplasmin	GGA ATA TGA GGG GGC CAT CTA TC
Ceruloplasmin L	Ceruloplasmin	GCA CGT CCA CTT CAT TAC CCA TGC C
alpha-fetoprot U	alpha-fetoprotein	GGC TGC TCC GCC ATC CAT CC
alpha-fetoprot L	alpha-fetoprotein	TTT TCC CCA TCC TGC AGA CAC TCC

### Mutations in c-MET

To investigate mutations in the tyrosine kinase domain of c-MET, a PCR was performed with two overlapping primer sets for this domain (Table 1), both resulting in an approximately 750 bp product. PCR conditions were as described above with an annealing temperature of 50°C. The products were sequenced using an ABI 3100 Genetic Analyzer (Applied Biosystems, Nieuwerkerk a/d IJssel, The Netherlands). Sequence analysis and alignments were performed with Lasergene software (DNASTAR Inc., Madison, WI).

### Samples for Real Time PCR

Quantitative gene expression measurements of the cHCC cell line were compared with a group of four healthy liver tissues. Liver biopsies from the healthy dogs, which included two Cairn terriers (breed of the donor dog), were obtained under local anesthesia with a 16G biopsy needle and immediately snap-frozen and stored at -70°C until further analysis.

### Quantitative measurements of mRNA levels of gene products involved in neoplastic growth and apoptosis

Real-Time PCR based on the high affinity double-stranded DNA-binding dye SYBR green I (SYBR^® ^green I, BMA, Rockland, ME) was performed in triplicate in a spectrofluorometric thermal cycler (iCycler^®^, BioRad). Per reaction, 1.67 μl of cDNA was used in a 50 μl volume containing 1 × manufacturer's buffer, 2 mM MgCl_2_, 0.5 × SYBR^® ^green I, 200 μM dNTP's, 0.4 μM of each oligonucleotide primer, 1.25 units of AmpliTaq Gold (Applied Biosystems), on 96-well iCycler iQ plates (BioRad). Primers (Table 2) were designed using PrimerSelect software (DNASTAR Inc.). All PCR protocols included 40 cycles consisting of denaturation at 95°C for 20 sec, annealing for 30 sec, and elongation at 72°C for 30 sec. Melt curves (iCycler, BioRad), gel electrophoresis, and sequencing were used to examine each sample for purity and specificity. For each experimental sample, the amount of the genes under study, and of the housekeeping genes HPRT and beta-actin, were determined from the appropriate standard curve in autonomous experiments. Results were normalized according to the average amount of housekeeping genes and the values divided by the normalized values of the healthy group to generate relative expression levels [5]. Statistical analysis was performed using the Student T-test, and the level of significance was set to a *p *value 0.05.

**Table 2:** Oligonucleotides for quantitative RT-PCR used in this study.

**Primer**	**Extension**	**Primer sequence (5'-3')**	**Tm**	**Product size (bp)**	**Accession Number**
HPRT	U	AGCTTGCTGGTGAAAAGGAC	56	100	L77488/ L77489
	L	TTATAGTCAAGGGCATATCC			
Beta-actin	U	GATATCGCCGCGCTCGTCGTC	55	350	Z70044
	L	GGCTGGGGTGTTGAAGGTCTC			
c-MET	U	TGTGCTGTGAAATCCCTGAATAGAATC	58	112	AB118945
	L	CCAAGAGTGAGAGTACGTTTGGATGAC			
PTEN	U	AGATGTTAGTGACAATGAACCT	64,5	110	U92435
	L	GTGATTTGTGTGTGCTGATC			
P27Kip	U	CGGAGGGACGCCAAACAGG	59	90	AY455798
	L	GTCCCGGGTCAACTCTTCGTG			
Bcl-2	U	TGGAGAGCGTCAACCGGGAGATGT	62	87	AB116145
	L	AGGTGTGCAGATGCCGGTTCAGGT			
Beta-catenin	U	ATGGGTAGGGCAAATCAGTAAGAGGT	64	107	AY485996
	L	AAGCATCGTATCACAGCAGGTTAC			
TGF-alpha	U	CCGCCTTGGTGGTGGTCTCC	63	122	AY458143
	L	AGGGCGCTGGGCTTCTCGT			
SOCS3	U	ACACCAGCCTGCGCCTCAAGACCT	63	119	AY485997
	L	CGCCTCGCCGCCCGTCA			
Collagen I	U	GTGTGTACAGAACGGCCTCA	61	111	AF056303
	L	TCGCAAATCACGTCATCG			
ODC	U	GTGGGCGATTGGATGCTCTTTG	59	111	BI395954
	L	TGTTGGCCCCGACATCACATAGTAG			

### Ethics

All our procedures concerning animal use were approved by the owners and by the Utrecht University's Ethical Committee, as required by the Dutch law. The animals received human care in line with the University's guidelines.

## Abbreviations

ALT – alanine aminotransferase; Akt/PKB – ser/thr protein kinase B (also PKB/Akt); AST – aspartate aminotransferase; CK7 – cytokeratin 7; GLDH – glutamate-lactate dehydrogenase; HCC – hepatocellular carcinoma; HepPar1 – hepatocyte paraffin-1; HGF – hepatocyte growth factor; HPRT – hypoxanthine phosphoribosyl transferase; MDCK – Madin-Darby canine kidney cells; ODC – ornithine decarboxcylase; P27KIP – kinase inhibitor protein 27 kDa; PTEN – phosphatase and Tensin homolog deleted on chromosome TEN; SOCS3 – suppressors of cytokine signalling type 3; TGF-alpha – transforming growth factor alpha.

## Author's contributions

SYB carried out the establishment, the biochemical and molecular characterization, and the mutations in c-Met study. BS carried out the quantitative RT-PCR, whereas JY and RK carried out the histomorphological part. TvdI performed the description of the initial tumor. SYB, HFE, TvdI, JR and LC participated in the study design and coordination of the study. All authors read and approved the final manuscript.
